# Incidents of Animal Life

**Published:** 1887-03-19

**Authors:** Charles Swainson

**Affiliations:** Rector of Old Charlton, Kent) book, "*The Folk-Lore and Provincial Names of British Birds*"


### INCIDENTS OF ANIMAL LIFE.

Collated from the Rev. Charles Swainson's (M.A., Rector of Old Charlton, Kent) book, "*The Folk-Lore and Provincial Names of British Birds.*"

### SUPERSTITIONS ABOUT BIRDS.

The Wren.

THE wren has borne the designation of king of birds from time immemorial. An account is given of the cruel custom of hunting the wren on St. Stephen's Day, once practised in various parts of Ireland, the Isle of Man, Pembrokeshire and Essex, and in France. There are several traditions as to the origin of this custom, one of them being that—"St. Stephen, when being brought to execution, was escaping from his sleeping gaolers, when a wren flew on the face of one of them and woke him." A newer version relates that on one occasion James II.'s forces were on the point of surprising King William's army early in the morning, when some wrens, attracted probably by the fragments of the preceding night's repast, alighted on the head of a drum which had served for a table, and the noise of their bills in the act of picking awoke the drummer, who instantly beat to arms, and saved William's army from defeat. The wren, accordingly, has ever since been a prime favourite with the Orange party. In the Isle of Man, the feathers of the wren have long been regarded as a charm against shipwreck. The redbreast is a favourite everywhere, and there are many superstitions as to the evils that will befall anyone who wilfully kills the bird.

The Swallow.

The heart of the swallow, worn round the neck, was supposed to render the wearer attractive: it was also good for strengthening the memory; while the present to a lady of a gold ring which had lain in a swallow's nest nine days, engendered love for the donor in her breast. Nothing is supposed to bring better luck to a house than for swallows to build their nests round it; or, on the other hand, be a worse omen than for the nests to be forsaken. The first sight of a swallow (like the first note of a cuckoo) is a matter of no small importance among the German peasants. In Westphalia, when a man sees the first swallow, he should look if there be a hair under his foot. If he finds one, his future wife's hair will be of the same colour. In Bohemia, if one swallow be seen by a maiden, she will be married during the year; if a pair first meet her eye, she will remain single. The sight of a sitting swallow is accounted fortunate; of one on the wing, the reverse. In the Mark, it is necessary to wash the face immediately after the first swallow has been seen; otherwise one must expect to be sunburnt and freckled during the year. But the swallow is viewed in another light. In some cases, and amongst some nations, particularly those belonging to the Celtic race, the reverence and respect in which the bird is held proceed from fear; and its influence upon mankind, instead of being propitious, is sinister and diabolical. Hence in Ireland, according to Dr. Whateley, it is called "devil's bird"; and the country people hold that there is a certain hair on everyone's head, which, if a swallow can pick off, the man is doomed to eternal perdition. So, too, in some parts of Scotland it is said to have a drop of the devil's blood in its veins, and in Caithness it is called "witch hag."

The Magpie.

The magpie is almost universally considered to be a bird of evil omen. "In Germany and the North witches often transform themselves into its shape or use it as their steed." In Scotland the magpie was sometimes called the "devil's bird," and was believed to have a drop of the devil's blood in its tongue. The country folk in Oldenburg consider the magpie to be so imbued with Satanic principles that, if a cross be cut on a tree in which the bird has built, she will forsake her nest at once. There are several reasons for this bird's bad reputation in the north of England. One of them is "because she was the only bird that would not go into the ark with Noah and his folk. She liked better to perch on the roof and jabber over the drowning world." The appearance of a magpie is, according to popular belief, something of mysterious import, and various practices are used in different localities to avert ill-luck. In some parts, however—in Shropshire, for instance, and throughout Norway—magpies are considered harbingers of good-luck As an illustration of the opposite lights in which the magpie is regarded, may be mentioned the belief in Tyrol that broth in which this bird is boiled will make him who drinks it crazy. On the other hand, the pastor in a village near Dresden is reported to have cured several epileptic patients by the same drink.

Crows and Ravens.

In folk-lore, the crow always appears as a bird of the worst and most sinister character, representing either death, or night, or winter. In German Switzerland it is believed that a crow perching on the roof of a house in which lies a corpse, is a sign that the soul of the dead is irrevocably damned. And in Sussex, its cry thrice repeated is considered a sure token of death. The raven lives to so great an age that the ancients believed the time allotted to it was twenty-seven times that of a man. Jewish authors tell strange stories of this bird: that it was originally white, and was turned black for its deceitful conduct; also that it flies crooked because it was cursed by Noah. There is a widespread belief that the appearance of the raven prognosticates death. In Denmark, its appearance in a village is considered an indication that the parish priest will soon die. In Andalusia, if it is heard croaking over a house, an unlucky day is expected; repeated thrice, it is a fatal presage; if, perching high, turning and croaking, a corpse will soon come from that direction. In many parts of Germany they are believed to hold the souls of the damned, sometimes to be the Evil One himself.

Fortunate Auguries.

The flesh of the lark was supposed by the old mediciners to strengthen the voice and increase its sweetness; while in Bohemia, its eggs are still believed to have the same property. To the kingfisher was ascribed by the ancients the power of enriching its possessors, of preserving peace and harmony in families, and imparting to the lady who wore its feathers additional grace and loveliness.

The Cuckoo.

Among the superstitions connected with the hearing of the cuckoo's first call are these. In the maritime Highlands and Hebrides, if the cuckoo is first heard by one who has not broken his fast, some misfortune is expected. Indeed, besides the danger, it is considered a reproach to one to have heard the cuckoo while hungry. In France, to hear the cuckoo for the first time fasting is to make the hearer an idle do-nothing for the rest of the year, or to numb his limbs for the same period. There is a similar belief in Somersetshire. In Northumberland, one is told if you are walking on a hard road when the cuckoo first calls that the ensuing season will be full of calamity: to be on soft ground is a lucky omen. In Westphalia, the peasants, on hearing the cuckoo for the first time, roll over and over on the grass in order to insure themselves against lumbago for the rest of the year. This is considered all the more likely to happen if the bird repeats his cry while they are on the ground. The Bohemians take the cuckoo for a transformed peasant-woman, who hid herself when she saw the Lord Jesus approaching, lest she should be obliged to give him a loaf. After He had gone, she put her head out of a window and cried, "Cuckoo!" whereupon she was at once changed into a bird.

Di meliora!

The common consent of all nations, we are told, has decided that the screech-owl is a bird of evil omen. An omelette made of the eggs of the long-eared owl is believed, in Belgium, to be a remedy for drunkenness. The pigeon is considered to be essentially a bird of death. Thus, if a white pigeon settles on a chimney, some one of the occupants of the house will pass away ere long; but should the bird enter and perch on the table, it is considered a less portentous omen, and to signify sickness. This is a widespread belief through England. It is a common idea that no one can die happily on a bed in which there is even a single pigeon's feather. There is a similar superstition about partridge feathers, and there is an old saying that he who is sprinkled with pigeon's blood will never die a natural death. The various legends in which the lapwing is mentioned show that it is almost universally held in bad esteem (except for its flesh and eggs). In the south of Scotland, the peasantry bear it a traditional antipathy, arising from the raids upon the Covenanters by troopers who were directed to their hiding-places by its cries of alarm. The sad wailing cry of the curlew, while on the wing in the dark, still nights of winter, is believed, in some parts of England, to be a death-warning, and is called the cry of the seven whistlers. This term is also applied to the redwing, the wild goose, and the plover. In Shropshire and Worcestershire, the seven whistlers are considered to be "seven birds, and the six fly about continually together looking for the seventh, and when they find him the world will come to an end."

